# Group 2 Innate Lymphoid Cells Are Critical for the Initiation of Adaptive T Helper 2 Cell-Mediated Allergic Lung Inflammation

**DOI:** 10.1016/j.immuni.2014.01.011

**Published:** 2014-03-20

**Authors:** Timotheus Y.F. Halim, Catherine A. Steer, Laura Mathä, Matthew J. Gold, Itziar Martinez-Gonzalez, Kelly M. McNagny, Andrew N.J. McKenzie, Fumio Takei

**Affiliations:** 1 Terry Fox Laboratory, British Columbia Cancer Agency, Vancouver, British Columbia V5Z 1L3, Canada; 2 Genetics Graduate Program, College for Interdisciplinary Studies, University of British Columbia, Vancouver, British Columbia V6T 1Z2, Canada; 3 Medical Research Council, Laboratory of Molecular Biology, Cambridge, Cambridgeshire CB2 0QH, UK; 4 Biomedical Research Centre, University of British Columbia, Vancouver, British Columbia V6T 1Z3, Canada; 5 Department of Pathology and Laboratory Medicine, University of British Columbia, Vancouver, British Columbia V6T 2B5, Canada

## Abstract

Naive CD4^+^ T cell differentiation into distinct subsets of T helper (Th) cells is a pivotal process in the initiation of the adaptive immune response. Allergens predominantly stimulate Th2 cells, causing allergic inflammation. However, why allergens induce Th2 cell differentiation is not well understood. Here we show that group 2 innate lymphoid cells (ILC2s) are required to mount a robust Th2 cell response to the protease-allergen papain. Intranasal administration of papain stimulated ILC2s and Th2 cells, causing allergic lung inflammation and elevated immunoglobulin E titers. This process was severely impaired in ILC2-deficient mice. Whereas interleukin-4 (IL-4) was dispensable for papain-induced Th2 cell differentiation, ILC2-derived IL-13 was critical as it promoted migration of activated lung dendritic cells into the draining lymph node where they primed naive T cells to differentiate into Th2 cells. Papain-induced ILC2 activation and Th2 cell differentiation was IL-33-dependent, suggesting a common pathway in the initiation of Th2 cell responses to allergen.

## Highlights

• ILC2-deficient mice have impaired Th2 cell responses to allergen
• Allergen-induced Th2 cell differentiation is dependent on ILC2-derived IL-13
• ILC2 activation by allergen requires IL-33 from epithelial cells
• ILC2-derived IL-13 promotes DC migration to lymph node

## Graphical Abstract

**Figure:**
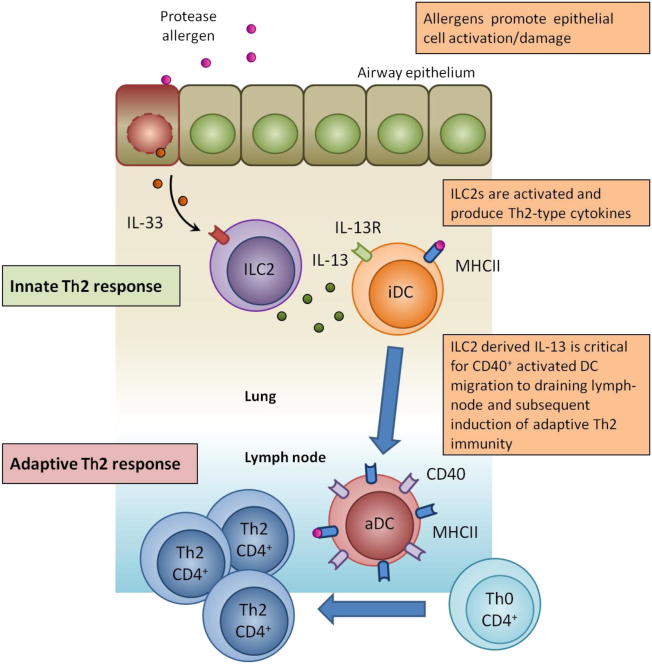

## Introduction

Allergy is one of the most common health problems in the industrialized world. A type 2 immune response is responsible for most allergen-induced inflammation at mucosal surfaces and is reflected in an overproduction of T helper 2 (Th2) cell-type (type 2) cytokines and immunoglobulin E (IgE) (Pulendran and Artis, 2012). Individuals might be sensitized to specific allergens, which stimulate naive CD4^+^ T cells to differentiate into Th2 cells. The reexposure of sensitized individuals to the same allergens causes a robust stimulation of memory Th2 cells that secrete the cardinal type 2 effector cytokines interleukin-4 (IL-4), IL-5, IL-9, and IL-13 (Kim et al., 2010, Lloyd and Hessel, 2010). In parallel, antigen crosslinking of IgE bound to FcεRI on mast cells and basophils leads to activation and degranulation, amplifying allergic inflammation of the affected tissues. Currently, the mechanisms by which allergens initiate the differentiation of naive CD4^+^ T cells into Th2 cells during the sensitization phase are not well understood. It is generally thought that the cytokine environment dictates the differentiation of naive CD4^+^ T cells into various populations of Th cells. IL-4 in particular is believed to be critical for Th2 cell differentiation, and binding to its receptor activates STAT6, which induces the expression of the key transcription factor GATA3 and drives the production of type-2 cytokines. However, the initial source of IL-4 responsible for the differentiation of naive CD4^+^ T cells into Th2 cells has been unclear because multiple cell populations, including natural killer T (NKT) cells, γδ T cells, basophils, dendritic cells (DCs), and naive CD4^+^ T cells can produce IL-4 (Weiss and Brown, 2001, Yamane and Paul, 2013). Moreover, Th2 cell differentiation can also be induced in vitro in the absence of exogenous IL-4 by IL-2, which induces IL-4Rα expression (Liao et al., 2008). Additionally, Th2 cell responses can be induced in vivo in IL-4- or IL-4R-deficient mice, indicating that an IL-4-independent pathway of Th2 cell differentiation exists. Currently, how IL-4-independent development of Th2 cells occurs is not well understood.

Notably, epithelial cell-derived cytokines, including IL-33, thymic stromal lymphopoietin (TSLP), and IL-25, are known to promote Th2 cell responses and allergic inflammation (Islam and Luster, 2012). The receptors for these cytokines are expressed by a variety of cell types including DCs, basophils, and NKT cells, but not naive CD4^+^ T cells. Mice deficient for the IL-33 receptor, ST2, produce reduced amounts of IL-4 and IL-5 in response to challenge with helminth antigen (Townsend et al., 2000) and IL-33 has been reported to activate DCs and induce allergic airway inflammation (Besnard et al., 2011). The stimulation of DCs (Zhou et al., 2005) and basophils (Siracusa et al., 2011) by TSLP is also thought to be critical for allergic inflammation. Nevertheless, the exact mechanisms by which these epithelial cell-derived cytokines promote Th2 cell differentiation are still unclear.

Group 2 innate lymphoid cells (ILC2s, previously termed natural helper cells, nuocytes, or Ih2 cells) (Spits et al., 2013), recently discovered in the gut (Moro et al., 2010, Neill et al., 2010, Price et al., 2010) and airway mucosa of mice (Chang et al., 2011, Halim et al., 2012a, Monticelli et al., 2011) and man (Mjösberg et al., 2011), are rapid and potent producers of the type 2 cytokines IL-5 and IL-13. With the discovery of ILC2s, we now understand that type 2 immunity comprises both innate and adaptive components. Papain, a protease known to be allergenic to humans and causes occupational asthma (Novey et al., 1979), is often used as a model allergen. Subcutaneous injection of papain into mice induces Th2 cell-mediated immunity (Tang et al., 2010). We have previously shown that intranasal administration of papain rapidly induces activation of lung IL-5 and IL-13-producing ILC2s, lung eosinophilia, and mucus hyperproduction in RAG-deficient mice. Thus, ILC2 activation can induce T cell- and IgE-independent acute allergic lung inflammation (Halim et al., 2012a). We also found that retinoic acid receptor related orphan receptor alpha (RORα) is critical for ILC2 development, and RORα-deficient Staggerer (*Rora*^sg/sg^) bone marrow (BM)-transplanted (BMT) mice are specifically deficient for ILC2s (Halim et al., 2012b, Wong et al., 2012). These mice fail to develop acute type 2 lung inflammation after sensitization with papain. Notably, *Rora*^sg/sg^ CD4^+^ T cells are not intrinsically impaired to develop into Th2 cells, and the observed defect in acute type 2 inflammation can be attributed to the lack of functional ILC2s in *Rora*^sg/sg^ BMT mice. Because ILC2s are a potent and early source of type 2 cytokines, we hypothesized that they could influence the downstream adaptive Th2 cell response. To test this hypothesis, we have examined the effects of ILC2-deficiency on Th2 cell responses to papain. Here we show that ILC2s were required for Th2-cell-mediated allergic lung inflammation. IL-13 produced by activated ILC2s was critical for promoting the migration of activated lung DCs to the draining lymph node (LN), where they induced the differentiation of naive CD4^+^ T cells into Th2 cells. Thus, our data reveal how innate ILC2 can play a critical role in the generation of adaptive Th2 cell responses to allergens.

## Results

### Protease-Allergen Papain Induced a Strong Innate and Adaptive Type-2 Immune Response

**Figure 1:**
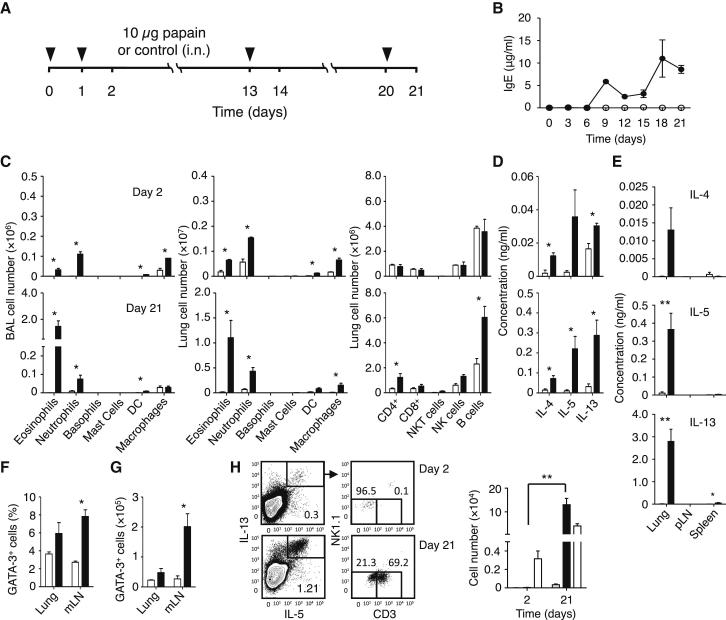
ILC2s and CD4^+^ T Cells Collaborated in Protease Allergen-Driven Innate and Adaptive Type-2 Lung Inflammation (A) Mice were sensitized by intranasal injection of 10 μg heat-inactivated (control) or active papain on days 0 and 1, followed by antigen challenge on days 13 and 20. (B) Serum concentrations of IgE were quantified on the days indicated (● papain, ○ control). (C–E) Mice injected with papain (▪) or control (□) were sacrificed on days 2, 14, and 21, followed by the quantification of myeloid populations in the bronchoalveolar lavage (BAL) and lung tissue by flow cytometry (C). Similarly, lung lymphoid populations were quantified. Cytokine concentrations in BAL were quantified by ELISA (D). Lymphocytes from indicated tissues were restimulated in vitro with recall antigen, followed by measurement for cytokine production (E). (F and G) Intracellular GATA3 was detected in CD4^+^ T cells from lung and mLN on day 21 following stimulation with papain (▪) or control (□). Shown are percent (F) and absolute numbers (G) of GATA3^+^ CD4^+^ T cells. (H) Lung cells were stained for intracellular IL-5 and IL-13 and analyzed by flow cytometry. The percentage of total IL-5^+^IL-13^+^ lung cells was measured in papain-treated or control mice on days 2 and 21. Double-positive cells were gated (left) and analyzed for surface expression of CD3 and NK1.1 to distinguish NK1.1^−^CD3^+^ T cells from NK1.1^−^CD3^−^ ILC2s (for detailed analysis, see Figure S1). Cells were first gated for live (eFluor780^−^) CD45^+^ cells. The absolute numbers of lung IL-5^+^IL-13^+^ ILC2s (□) or CD4^+^ T cells (▪) in papain-treated mice, and IL-5^+^IL-13^+^ CD4^+^ T cells in control mice () were calculated on days 2 and 21. Data are representative of at least three independent experiments. Mean ± SEM in (B)–(H), mean percent gated in H, ^∗^ = p ≤ 0.05 ^∗∗^ = p ≤ 0.01 (two-tailed Student’s t test). See also Figure S1.

Mice were sensitized to Th2 cell-mediated allergic responses by the intranasal administration of papain (or heat-inactivated papain as a control) on days 0 and 1, followed by two challenges on days 13 and 20 (Figure 1A). Serum IgE titers increased upon sensitization and subsequent challenges (Figure 1B). Leukocytes, including eosinophils and neutrophils, rapidly infiltrated into bronchoalveolar lavage (BAL) and lung tissue following sensitization, and low amounts of type 2 cytokines were detected in the BAL on day 2 (Figures 1C and D). Activated ILC2s rather than Th2 cells likely mediated these early responses to papain, since similar responses were observed in *Rag1*^*−/−*^ mice that have ILC2s but lack T and B cells (see Figures S1A–S1C available online). The subsequent challenge of papain-sensitized mice induced the infiltration of substantially higher numbers of eosinophils in the BAL (9-fold increase, p = 0.02) and the lung (15-fold increase, p = 0.005) as well as significantly higher concentrations of type 2 cytokines in the BAL (p = 0.0002) on day 21 (Figures 1C and D). Th2 cells likely mediated these latter responses to papain since *Rag1*^−/−^ mice did not show the augmented immune response (Figures S1A–S1C). In wild-type (WT) mice, the draining mediastinal lymph nodes (mLN) were enlarged with substantially increased numbers of CD4^+^ T cells and B cells on day 21 (data not shown). Notably, basophils and mast cells were not detected in substantial numbers at the time-points investigated.

To confirm the induction of papain-specific Th2 cells, we isolated lymphocytes from the lungs, peripheral (inguinal) LNs (pLN), and spleens of papain-treated or control mice on day 21 and restimulated them with recall-antigen (Figure 1E) or immobilized anti-CD3ε plus anti-CD28 (Figure S1D). The in vitro-stimulated lung lymphocytes from papain-treated, but not control, mice produced IL-4, IL-5, and IL-13. We also analyzed the expression of GATA3, a critical transcription factor for Th2 cell differentiation (Zheng and Flavell, 1997), in CD4^+^ T cells on day 21 by flow cytometry (Figures S1E and S1F). Both the percentages and the absolute numbers of GATA3^+^ CD4^+^ T cells were higher in the mLNs of the papain-treated mice compared to control mice (Figures 1F and 1G).

To identify the cellular source of type 2 cytokines at various time points, we stained for intracellular IL-5 and IL-13 and cell-type-specific surface markers in the lung (Figure 1H) and mLN (Figure S1I). As expected from our previous studies (Halim et al., 2012a), IL-5^+^IL-13^+^ cells on day 2 were primarily ILC2s. On day 21, the majority of IL-5^+^IL-13^+^ cells were CD4^+^ T cells (Figure 1H), while ILC2s also expanded and comprised approximately 30% of IL-5^+^IL-13^+^ cells in the lung on day 21 (Figure 1H; Figures S1J and S1K). Thus, intranasal administration of papain initially stimulated ILC2s and also induced Th2 cell differentiation, whereas the subsequent challenges with papain stimulated primed Th2 cells, resulting in type 2 cytokine production, increased IgE titers, and eosinophilic lung inflammation.

### ILC2s Were Required for Induction of the Adaptive Type-2 Immune Response

**Figure 2:**
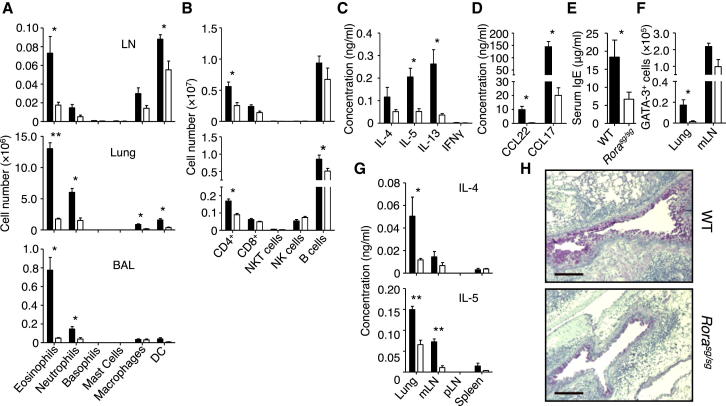
ILC2-Deficient Mice Were Incapable of Mounting an Effective Th2 Cell Response and Had Greatly Reduced Type 2 Lung Inflammation (A–G) WT (▪) and *Rora*^sg/sg^ (□) BMT mice were treated with papain and analyzed on day 21 for myeloid cell numbers in the mLN, lung, and BAL (A). Lymphoid cell numbers were measured in the mLN and lung (B). The BAL was analyzed for concentrations of cytokines (C) and chemokines (D). Serum IgE concentrations were measured (E). The absolute number of GATA3^+^ CD4^+^ T cells was determined in the lung and mLN (F). Cells were restimulated with recall antigen, followed by an analysis for cytokine production in culture supernatant (G). (H) Formalin-fixed tissue was analyzed by histology for mucus production in PAS stained sections. Data are representative of at least three independent experiments. Scale bar represents 100 μm. Mean ± SEM in (A)–(G), ^∗^ = p ≤ 0.05 ^∗∗^ = p ≤ 0.001 (two-tailed Student’s t test). See also Figure S2.

On the basis of the above results, we hypothesized that ILC2s promote Th2 cell differentiation in papain-treated mice. To test this, we investigated the effects of ILC2-deficiency on Th2 cell responses to papain. We have previously shown that the transcription factor RORα is required for ILC2 development and that transplantation of BM from RORα mutant *Rora*^sg/sg^ mice into irradiated WT mice generates ILC2-deficient mice (Halim et al., 2012b, Wong et al., 2012). Importantly, *Rora* is not highly expressed in other hematopoietic cells, including naive and memory T cells (Figure S2A). We have also previously shown that *Rora*^sg/sg^ CD4^+^ T cells have no inherent defect in their capacity to differentiate into Th2 cells in vitro, indicating that RORα is not intrinsically required for Th2 cell differentiation (Halim et al., 2012b). Thus, WT bone-marrow-transplanted (WT BMT) and ILC2-deficient *Rora*^sg/sg^ BMT mice were administered papain as in Figure 1A, and Th2 cell responses were compared after the second challenge (day 20). *Rora*^sg/sg^ BMT mice had strikingly fewer eosinophils in the BAL, lung, and mLN, and fewer neutrophils, DCs, and CD4^+^ T cells in the lung parenchyma and mLN than WT BMT mice (Figures 2A and 2B). The inability of *Rora*^sg/sg^ BMT mice to mount strong Th2 cell responses to papain was further supported by the substantially lower amounts of type 2 cytokines and the Th2 cell-associated chemokines CCL22 and CCL17 (Bromley et al., 2008) in the BAL (Figures 2C and 2D) as compared to controls. Papain-challenged *Rora*^sg/sg^ BMT mice also displayed substantially reduced levels of IgE in the serum (Figure 2E) and substantially lower numbers of GATA3^+^ CD4^+^ T cells in the lung compared to WT BMT mice (Figure 2F; Figure S2B). Furthermore, the in vitro restimulation of lymphocytes from the lung and mLN of *Rora*^sg/sg^ BMT mice with recall-antigen resulted in substantially less type 2 cytokine production than those from WT BMT mice (Figure 2G). Histological analyses also revealed substantially less mucus production and inflammation in the lungs of *Rora*^sg/sg^ BMT mice than WT BMT mice (Figure 2H; Figures S2C–S2E). Together, these results clearly demonstrate a profound defect in Th2 cell immunity in the absence of ILC2s.

### IL-4 Was Not Required for an Efficient Adaptive Th2 Cell Immune Response to Inhaled Protease Allergen

**Figure 3:**
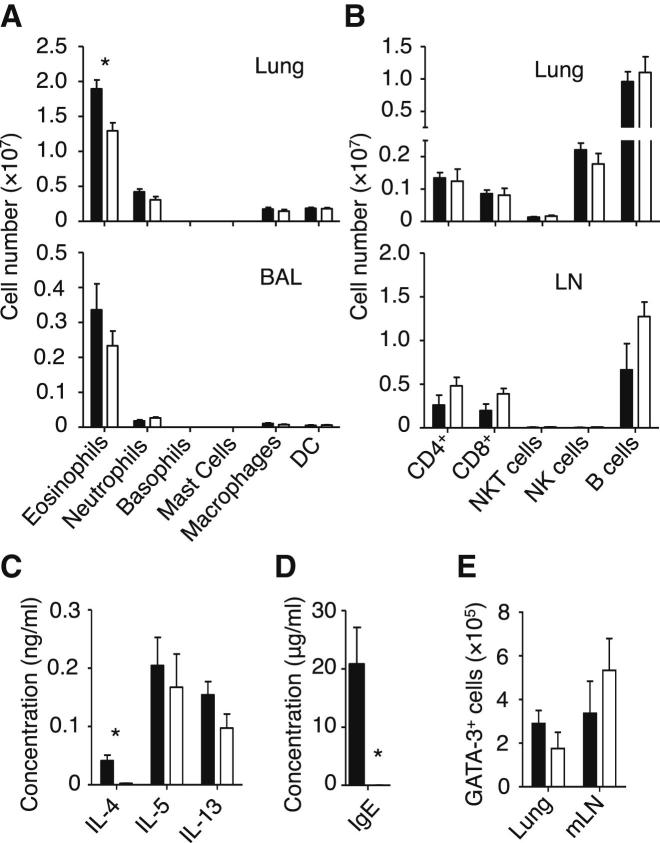
IL-4 Played a Nonessential Role in the Allergic Response to Protease Allergen and Induction of Type 2 Lung Inflammation (A–E) WT (▪) and *Il4*^*−/−*^ (□) mice were treated with papain and analyzed on day 21 for myeloid cell numbers in the lung and BAL (A), lymphoid cell numbers in the lung and mLN (B), cytokine concentrations in the BAL (C), and IgE concentration in serum (D). Intracellular GATA3 was detected in CD4^+^ T cells from lung and mLN on day 21 following stimulation with papain, and absolute numbers of GATA3^+^ CD4^+^ T cells were calculated (E). Data are representative of three independent experiments. Mean ± SEM in (A)–(E), ^∗^ = p ≤ 0.05 (two-tailed Student’s t test). See also Figure S3.

Because IL-4 is thought to play a critical role in the differentiation of naive CD4^+^ T cells into Th2 cells (Pulendran and Artis, 2012), the above results suggested that ILC2s might be a source of IL-4 in papain-treated mice. However, purified ILC2s produced large amounts of IL-5 and IL-13 but very little IL-4 (Figures S3A and S3B). To clarify the role of IL-4 in papain-induced Th2 cell responses, we tested the activation of Th2 cells in *Il4*^*−/−*^ mice. Unexpectedly, *Il4*^*−/−*^ mice mounted a strong Th2 cell response to papain and they had comparable levels of eosinophils in the BAL as WT mice, although lung eosinophil numbers were slightly lower (Figure 3A). They also exhibited no substantial difference in the numbers of CD4^+^ T cells in the lung and mLN (Figure 3B), the amounts of IL-5 and IL-13 in the BAL (Figure 3C), or the number of GATA3^+^ Th2 cells in the lung and mLN (Figure 3E; Figure S3C) when compared to WT mice. As expected, *Il4*^*−/−*^ mice had no detectable IL-4 in the BAL (Figure 3C), and no detectable serum IgE (Figure 3D), which is known to be IL-4-dependent (Finkelman et al., 1988). Thus, IL-4 was dispensable for papain-induced Th2 cell generation, suggesting that ILC2s promote Th2 cell responses by an IL-4-independent mechanism.

### ILC2s Were Instrumental for Induction of Th2 Cells in the mLN

**Figure 4:**
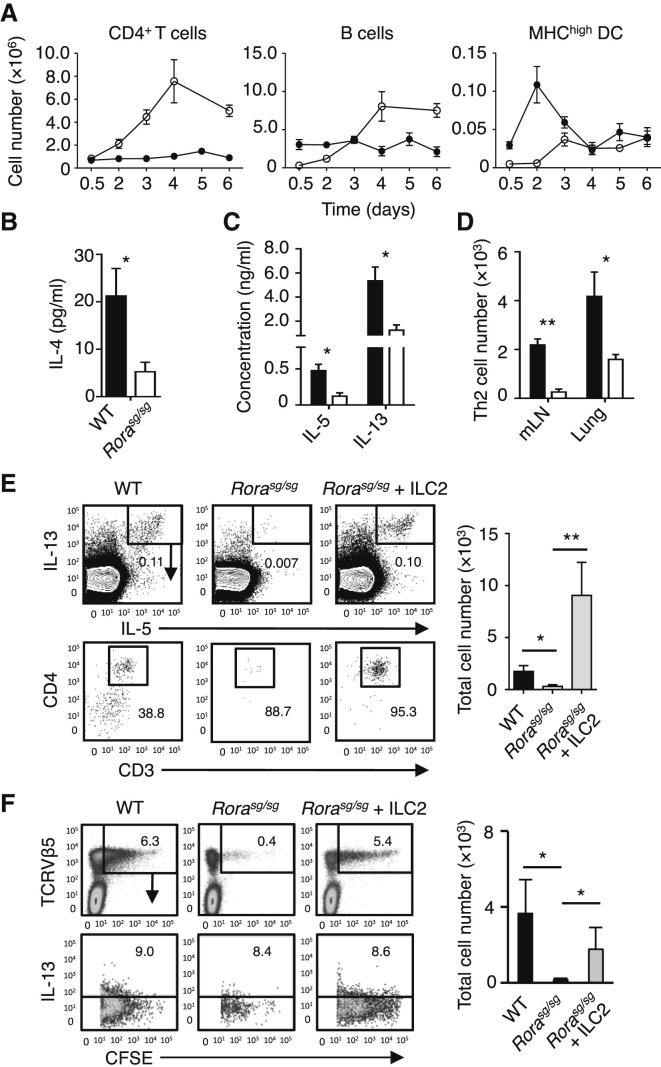
ILC2 Were Instrumental for Induction of Th2 Cells in the mLN (A) WT mice received intranasal papain administration on days 0 and 1, and the absolute numbers of CD4^+^ T cells, B cells, and MHC^hi^ DCs were measured in the lung (●) or mLN (○) at various time-points by flow cytometry. (B and C) On day 6, cells from papain-treated WT (▪) or *Rora*^sg/sg^ (□) BMT mouse mLN were restimulated in αCD3ε and αCD28 mAb coated wells, followed by analysis for cytokine production in culture supernatant. (D) Total numbers of IL-5^+^IL-13^+^ CD3^+^CD4^+^ Th2 cells from papain treated WT (▪) or *Rora*^sg/sg^ (□) BMT mouse lung and mLN were measured on day 6. (E) WT BMT, *Rora*^sg/sg^ BMT, or ILC2-transplanted *Rora*^sg/sg^ BMT mice were stimulated with papain on days 0 and 1, and mLN lymphocytes were analyzed for intracellular IL-5 and IL-13 on day 6 by flow cytometry (top plots). Gated IL-5^+^IL-13^+^ lymphocytes were analyzed for CD3^+^CD4^+^ cells (bottom plots), and the total numbers of Th2 cells in the mLN were calculated (bar graph). (F) CFSE-labeled CD4^+^ T cells from OT-II mice were transplanted into WT BMT and *Rora*^sg/sg^ BMT mice (±ILC2 transplantation), followed by intranasal injections of rmIL-33 + OVA on days 0 and 1. TCRVβ5^+^CFSE^+^ OT-II T cells in the mLN (top plots) were analyzed for intracellular IL-13 (bottom plots) by flow cytometry on day 6, and the total numbers of IL-13^+^ OT-II T cells in the mLN were calculated (bar graph). Data are representative of at least three independent experiments. Mean ± SEM in (A)–(F), mean percent gated in (E) and (F), ^∗^ = p ≤ 0.05 ^∗∗^ = p ≤ 0.01 (two-tailed Student’s t test). See also Figure S4.

To elucidate how ILC2s promote Th2 cell-mediated allergic responses to papain, we analyzed the initiation of Th2 cell differentiation from naive CD4^+^ T cells (Figure S4A). Following papain treatment on days 0 and 1, the numbers of B cells and CD4^+^ T cells in mLN steadily increased until day 4 (Figure 4A). The induction of Th2 cells was observed by day 6 via intracellular staining for type 2 cytokines in CD4^+^ T cells (data not shown). CD11c^+^MHCII^hi^ activated DCs in the lung rapidly increased in number, followed by their increase in the mLN (Figure 4A; Figures S4B and S4C). Thus, papain-activated lung DCs likely migrated into the draining mLN where they stimulated naive CD4^+^ cells. We then compared Th2 cell generation in the mLN of WT BMT and *Rora*^sg/sg^ BMT mice 6 days after the initial papain administration. The in vitro restimulation of mLN lymphocytes resulted in substantially lower IL-4, IL-5, and IL-13 production by *Rora*^sg/sg^ BMT mouse lymphocytes compared to WT controls (Figures 4B and 4C). Intracellular cytokine staining also demonstrated that the numbers of CD4^+^ Th2 cells expressing IL-5 and IL-13 in the mLNs and lungs of *Rora*^sg/sg^ BMT mice were substantially lower than those of WT BMT mice on day 6 (Figure 4D). Similar results were obtained when mice were stimulated with house dust mite (HDM) or a fungal protease-allergen (Figure S4D). Importantly, the adoptive transfer of ILC2s into papain-treated *Rora*^sg/sg^ BMT mice restored Th2 cell generation (Figures 4E; Figure S4E). These results showed that ILC2s were critical for the differentiation of naive CD4^+^ T cells into Th2 cells.

Lung ILC2 activation is contingent on stimulation via IL-33, an alarmin produced in response to a broad range of allergens (Hardman et al., 2013). We found that papain-driven IL-13 production from ILC2, eosinophilic lung inflammation, and Th2 cell differentiation were all impaired in intranasally challenged IL-33-deficient mice (Figures S4F-H). Moreover, intranasal administration of IL-33 alone directly stimulated lung ILC2s without papain treatment (Figure S4I) as reported (Barlow et al., 2012). We then intranasally administered ovalbumin (OVA) antigen together with IL-33 to WT BMT or *Rora*^sg/sg^ BMT mice, which were also intravenously injected with carboxyfluorescein succinimidyl ester (CFSE)-labeled CD4^+^ T cells from the OVA-specific T cell receptor transgenic OT-II mice. Very few OT-II T cells (CFSE^+^) were recovered in the mLNs of *Rora*^sg/sg^ BMT mice as compared to WT, and the numbers of IL-4^+^ IL-13^+^ Th2 cells generated in *Rora*^sg/sg^ BMT mice were substantially lower than those in WT BMT mice. Moreover, the impaired Th2 cell differentiation was rescued by ILC2 transplantation (Figure 4F). These results demonstrate that IL-33-mediated ILC2 activation was critical for effective Th2 cell differentiation, likely in response to the intranasal administration of a broad range of antigens.

### IL-13 from ILC2 Promoted Th2 Cell Differentiation in the Draining LN

**Figure 5:**
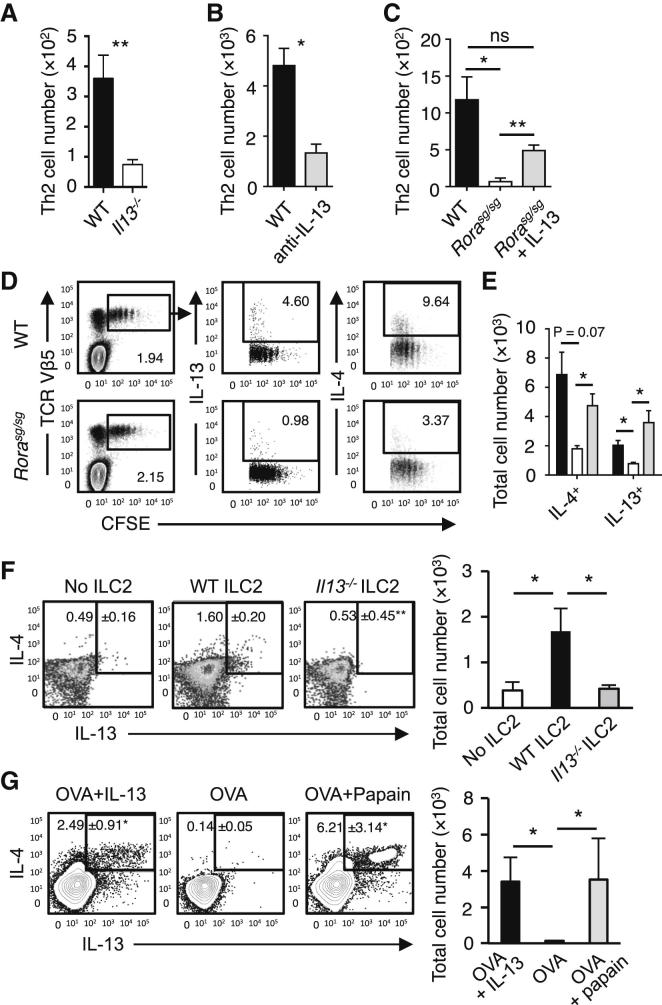
IL-13 Was Required for Induction of Th2 Cells (A) WT (▪) and *Il13*^*−/−*^ (□) mice were treated with papain on days 0 and 1, and the total numbers of IL-5^+^ CD3^+^CD4^+^ Th2 cells in the mLN on day 6 were measured. (B) IL-5^+^IL-13^+^ CD3^+^CD4^+^ Th2 cells were quantified on day 6 after papain stimulation on days 0 and 1 in WT mice (▪) or mice treated with anti-IL-13 mAb (). (C) The induction of Th2 cells in WT BMT mice stimulated with papain (▪) was compared to *Rora*^sg/sg^ BMT mice stimulated with papain (□) or papain + rmIL-13 (). The total numbers of IL-5^+^IL-13^+^ CD3^+^CD4^+^ Th2 cells in the mLN were quantified on day 6. (D) CFSE-labeled CD4^+^ OT-II cells were transplanted into WT and *Rora*^sg/sg^ BMT mice, followed by treatment with papain + OVA. On day 6, mLN cells were analyzed by flow cytometry for CD4^+^TCRVβ5^+^CFSE^+^ OT-II T cell proliferation and intracellular IL-4 and IL-13. (E) WT (▪) and *Rora*^sg/sg^ (□) were treated as in (D). Some of the latter mice also received rmIL-13 injection (). OT-II T cells positively stained for intracellular IL-4 or IL-13 were quantified and the total numbers of cytokine positive OT-II in the mLN were calculated. (F) *Rora*^sg/sg^ BMT mice were transplanted with CFSE-labeled CD4^+^ OT-II cells plus none, WT, or *Il13*^*−/−*^ ILC2s. These mice were treated on days 0 and 1 with papain, and CD3^+^CD4^+^TCRVβ5^+^CFSE^+^ OT-II T cells in the mLN were analyzed for intracellular IL-4 and IL-13 (plots) on day 6. The total numbers of IL-4^+^IL-13^+^ OT-II T cells in the mLN were calculated (bar graph). (G) CFSE-labeled CD4^+^ OT-II cells were transplanted into WT mice. The mice received intranasal administration of OVA + rmIL-13, OVA alone, or OVA + papain on days 0 and 1. CD3^+^CD4^+^TCRVβ5^+^CFSE^+^ cells were gated (data not shown) and analyzed for intracellular IL-4 and IL-13 on day 6 (plots). The total numbers of IL-4^+^IL-13^+^ OT-II T cells in the mLN were calculated (bar graph). Data are representative of at least three independent experiments. Mean ± SEM in (A)–(C) and (E)–(G), mean percent gated in (D), (F), and (G), ^∗^ = p ≤ 0.05 ^∗∗^ = p ≤ 0.01, ns = not significant (two-tailed Student’s t test). See also Figure S5.

The above results showed that ILC2s promoted Th2 cell differentiation while producing a large amount of IL-13 but little IL-4. Therefore, we tested whether IL-13 is involved in Th2 cell differentiation in papain-treated mice. Intranasal papain treatment of IL-13-deficient mice generated substantially fewer Th2 cells (IL-5^+^ CD4^+^ T cells) in the mLN compared to WT mice (Figure 5A). Moreover, IL-13 neutralization also inhibited Th2 cell differentiation in papain-treated WT mice (Figure 5B), whereas IL-13 injection enabled the generation of Th2 cells in papain-treated *Rora*^sg/sg^ BMT mice (Figure 5C).

The role of ILC2-derived IL-13 for Th2 cell differentiation was confirmed in a TCR-transgenic model, in which CFSE-labeled OT-II T cells were injected into WT or *Rora*^sg/sg^ BMT mice. The mice then received intranasal administration of OVA and papain, and OT-II T cells in the mLN were analyzed 6 days later. Although OT-II T cells proliferated in both WT and *Rora*^sg/sg^ BMT mice, the differentiation of naive OT-II T cells into Th2 cells was substantially impaired in *Rora*^sg/sg^ BMT mice (Figure 5D). Notably, this impaired Th2 cell differentiation in *Rora*^sg/sg^ BMT mice was rescued by the intranasal injection of IL-13 (Figure 5E). WT ILC2 transplantation also rescued Th2 cell production whereas IL-13-deficient ILC2s did not (Figure 5F). Interestingly, the intranasal administration of OVA alone, without papain, into WT mice that were injected with CD4^+^ OT-II T cells did not induce detectable Th2 cell differentiation in mLN, whereas the coadministration of OVA plus IL-13 efficiently induced Th2 cell differentiation of OT-II T cells (Figure 5G).

Intracellular staining for IL-13 after papain stimulation identified ILC2 as the primary cellular source, and ILC2-deficient mice did not produce IL-13 during the acute inflammatory response (Halim et al., 2012a, Halim et al., 2012b). Analysis of *Il13*^egfp/+^ reporter mice also confirmed that ILC2s are the predominant *Il13* expressing cells in papain-treated lungs (Figure S5A). Together, these results indicated that ILC2-derived IL-13 was critical for the differentiation of Th2 cells.

### IL-13 Promoted CD40^+^ DC Migration to the Draining LN

**Figure 6:**
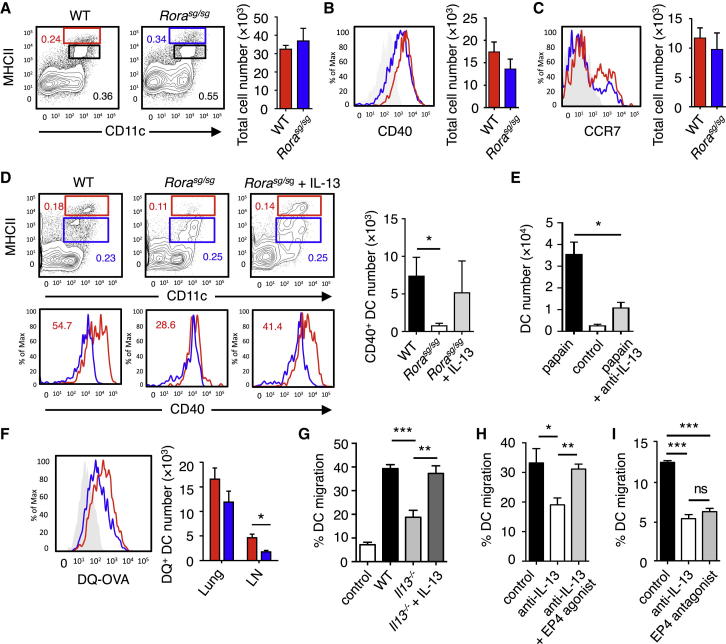
ILC2-Derived IL-13 Promoted DC Migration to the Draining LN (A–C) Lungs from WT (red) and *Rora*^sg/sg^ (blue) BMT mice treated on days 0 and 1 with papain were analyzed on day 3 for DC activation. CD11c^+^MHCII^hi^ activated DCs were detected by flow cytometry and the total numbers in the lung calculated (A). Activated DCs in (A) were analyzed for CD40 (B) and CCR7 (C) expression. The red histograms represent WT BMT DCs, the blue histograms represent *Rora*^sg/sg^ BMT and the solid gray histograms show the FMO control. Bar graphs are color-coded in the same way show the total numbers of DCs in the lung. (D) DCs in the mLN of WT and *Rora*^sg/sg^ BMT mice (±rmIL-13 injection) were analyzed on day 3. Activated CD11c^+^MHCII^hi^ DCs gated by red boxes and nonactivated DCs gated by blue boxes (top plots) were analyzed for CD40 expression (bottom histograms, color-coded in the same way). Number in histogram shows the percent CD40^+^CD11c^+^ cells. Absolute numbers of CD11c^+^MHCII^hi^ CD40^+^ cells in mLN were calculated (bar graph). (E) WT mice treated with papain (▪), papain + anti-IL-13 mAb (), or control (□) were analyzed for absolute numbers of activated DCs in mLNs on day 3 as in (D). (F) Traceable DQ-OVA was injected together with papain into WT (red) or *Rora*^sg/sg^ (blue) BMT mice, followed by an analysis for DQ-positive CD11c^+^MHCII^hi^ DCs in the lung and mLN on day 3 (histograms), and the total numbers of DQ^+^ DCs were calculated (bar graph). (G) Lung tissue explants were made from papain-stimulated WT, *Il13*^*−/−*^, or *Il13*^*−/−*^ + rmIL-13 mice on day 3. Tissue explants were cultured in transwell inserts and exposed to a CCL21 chemokine gradient for 14 hr. WT lung explants without a CCL21 gradient were used as control. The total number of activated DCs was counted in both top and bottom compartments by flow cytometry, followed by a calculation of percent DC migration. (H) Lung tissue explants from naive WT mice were stimulated ex vivo with papain for 14 hr in the presence of anti-IL-13 neutralizing antibody ± EP4-agonist or PBS control. The percent of DC migration toward a CCL21 chemokine gradient was calculated. (I) Lung explants were prepared, treated with papain in the presence of PBS (control), anti-IL-13 or EP4 antagonist, and DC migration was analyzed as in (H). Data are representative of at least three independent experiments. Mean ± SEM in (A)–(I), mean percent gated in (A) and (D), ns = not significant ^∗^ = p ≤ 0.05, ^∗∗^ = p ≤ 0.01 ^∗∗∗^ = p ≤ 0.001 (two-tailed Student’s t test). See also Figure S6.

We detected the IL-13 receptor on activated DCs, but not on CD4^+^ T cells (Figure S6A), and exogenously added IL-13 did not have a direct effect on in vitro Th2 cell differentiation (Figure S6B). Because DCs are known to play a critical role in Th2 cell induction to inhaled allergens (Kool et al., 2012), we hypothesized that ILC2-derived IL-13 might influence DC function. We first compared DCs in the lungs of WT mice treated with papain and those treated with heat-inactivated papain and found no differences in the expression of CD44, CD86, OX40L, and ICOSL (data not shown). We then analyzed DCs in the lungs and mLNs of WT and *Rora*^sg/sg^ BMT mice treated with papain. No substantial differences in the number of activated DCs or their phenotype (CD40 and CCR7 expression) was observed in the lung between WT BMT and *Rora*^sg/sg^ BMT mice (Figures 6A-6C). In contrast, a striking difference in the mLN DC populations was seen between WT BMT and *Rora*^sg/sg^ BMT mice. More than 50% of activated DCs in the mLNs of WT BMT mice expressed CD40, which has been shown to be critical for efficient Th2 cell differentiation (Jenkins et al., 2008, MacDonald et al., 2002). *Rora*^sg/sg^ BMT mice showed substantially reduced CD40^+^ DC numbers in the mLN (Figure 6D). Importantly, this impairment was rescued by IL-13 injection (Figures 6D), whereas IL-13 neutralization substantially reduced CD40^+^ DCs in the mLN of papain-treated WT mice (Figure 6E). We also coadministered papain and the synthetic antigen DQ-OVA, which becomes fluorescent upon processing by antigen-presenting cells, and analyzed antigen processing by DCs. The processing of antigen by lung DCs was not affected in *Rora*^sg/sg^ BMT mice, whereas the trafficking of DQ-labeled cells to the draining LN was substantially reduced (Figure 6F). These data suggested that ILC2-derived IL-13 might be critical for the migration of activated antigen-licensed CD40^+^ DCs from the lung to the draining mLN in papain-treated mice.

To further investigate the effect of IL-13 on DC migration, we prepared lung tissue explants from papain-treated WT or *Il13*^*−/−*^ mice and tested the migration of lung tissue-resident DC toward a gradient of the DC chemokine CCL21 in transwell cultures (Figures S6C and S6D). CCL21 (or CCL19) signaling via CCR7 is critical for DC migration to secondary lymphoid organs (Förster et al., 1999). DCs in lung tissue explants prepared from papain-treated WT mice demonstrated robust specific chemotaxis in the presence of CCL21, whereas IL-13 had no effect on its own (Figures S6D and S6E). The migration of *Il13*^*−/−*^ mouse lung DCs toward CCL21 was substantially lower than that of WT DCs but was rescued by the administration of recombinant IL-13 into the mice (Figure 6G).

It has been reported that prostaglandin E2 (PGE_2_) signaling via its receptor, EP4, is necessary to sensitize CCR7^+^ DCs to a CCL21 chemokine gradient (Kabashima et al., 2003, Luft et al., 2002, Scandella et al., 2002). IL-13 is also known to induce PGE_2_ production in DCs and macrophages (Legler et al., 2006, Rey et al., 1999). Indeed, IL-13 stimulation of lung leukocytes induced PGE_2_ production (Figure S6F). Gene-expression analysis indicated high expression of *Ptger4* (EP4) by naive lung DCs (Figure S6G). Furthermore, our flow-cytometric analyses showed that intranasal IL-13 treatment of mice upregulated EP4 expression on lung DCs as compared to control PBS-treated mice (Figure S6H). Thus, we tested whether EP4 was involved in DC migration in our system by using lung explant cultures of naive WT mice. The in vitro DC migration was substantially inhibited by IL-13 neutralization but was rescued by simultaneously adding an EP4-agonist (Figure 6H). EP4-antagonist also mimicked IL-13-neutralization and substantially inhibited DC migration (Figure 6I). Overall, these results suggested that IL-13 influenced lung DC migration, in part by modifying the PGE_2_-EP4 pathway.

The migration of activated DCs to the draining LN is thought to be essential for the induction of Th2 cell differentiation (Phythian-Adams et al., 2010, van Rijt et al., 2005). Indeed, papain-stimulated mLN DCs efficiently induced Th2 cell differentiation in vitro (Figure S6I). Furthermore, papain-induced Th2 cell-mediated allergic lung inflammation was impaired in the LN-deficient *Rag2*^*−/−*^*Il2rg*^*−/−*^ mice transplanted with WT or *Rora*^sg/sg^ BM (Figures S6J–S6M). Thus, the migration of activated lung DCs to the mLN was critical for the initiation of Th2 cell differentiation induced by inhaled allergens.

## Discussion

By using ILC2-deficient mice generated by transplanting BM of the *Rora* mutant Staggerer mice into irradiated recipients, we have demonstrated that ILC2s were required for efficient Th2 cell-mediated allergic lung inflammation induced by repeated intranasal administration of the protease-allergen papain. The inability of ILC2-deficient mice to mount strong allergic lung inflammation in response to intranasal papain was due to impaired Th2 cell differentiation in the draining mLN, and could be rescued in vivo by reconstituting ILC2s. Therefore, ILC2s played a critical role in the differentiation of naive CD4^+^ T cells into Th2 cells. Although IL-4 is thought to be critical for Th2 cell differentiation, the Th2 cell-promoting effect of ILC2s appeared to be mediated by IL-13 rather than IL-4, because IL-4-deficient mice mounted a potent Th2 cell-mediated response to papain, whereas IL-13-deficient mice did not. Furthermore, the intranasal administration of IL-13 into ILC2-deficient mice enabled these mice to mount a normal Th2 cell response to papain, whereas IL-13 neutralization in WT mice inhibited the papain-induced Th2 cell response. The transplantation of IL-13-deficient ILC2s into ILC2-deficient mice, unlike WT ILC2 transplantation, did not rescue this impaired Th2 cell differentiation, and the intranasal administration of IL-13 and OVA without papain into WT mice also promoted OVA-specific OT-II T cell differentiation into Th2 cells. Taken together, these results indicate that ILC2-derived IL-13 was critical for the differentiation of naive CD4^+^ T cells into Th2 cells in the mLN.

IL-13 is known to be a pro-Th2 cytokine (Wynn, 2003). The neutralization of IL-13 inhibits asthma symptoms in OVA-sensitized and challenged mice (Grünig et al., 1998), and IL-13-deficient mice have an impaired Th2 cell response to parasite infections (McKenzie et al., 1998). However, the source of IL-13 and the mechanisms by which IL-13 promotes Th2 cell differentiation have remained unclear. In the current study with papain-treated mice, ILC2s were the main source of IL-13. Although basophils and mast cells are also known to be capable of producing IL-13 (Kroeger et al., 2009), they were undetectable in papain-treated mouse lungs or mLNs. The receptor for IL-13 consists of IL-4Rα and IL-13Rα (Mentink-Kane and Wynn, 2004). DCs expressed IL-13Rα1, which was undetectable on naive CD4^+^ T cells. IL-13 also had no effect on Th2 cell differentiation in vitro (Figure S6, also [McKenzie et al., 1998]), indicating that IL-13 is unlikely to act directly on naive CD4^+^ T cells. Instead, DCs seemed to be the main target of ILC2-derived IL-13 in papain-treated mice. DCs in the draining mLN of WT and ILC2-deficient mice treated with papain differed substantially in their expression of the costimulatory ligand CD40. The effects of IL-13 administration on ILC2-deficient mice and IL-13 neutralization in WT mice indicated that ILC2-derived IL-13 was likely responsible for CD40^+^ DCs in the mLN. However, ILC2-derived IL-13 did not appear to directly induce CD40 expression on DCs, because DCs in the lungs of WT and ILC2-deficient mice treated with papain did not differ substantially in their expression of CD40 or their processing of antigen (DQ-OVA). Instead, IL-13 appeared to stimulate the migration of activated DCs expressing CD40 from the lung to the mLN.

Our in vitro studies with *Il13*^*−/−*^ lung explants and IL-13-neutralization demonstrated that IL-13 plays an important role for lung DC migration toward the DC chemokine CCL21, which binds to its receptor, CCR7 (Förster et al., 1999). Although CCR7 is known to be critical for DC migration to the draining LN, WT and ILC2-deficient mice treated with papain did not differ in terms of lung DC CCR7 expression. It has been reported that CCR7 expression alone is insufficient for skin DC migration toward a CCL21 chemokine gradient, requiring additional stimuli, which can be provided by the ligation of the PGE_2_-receptor EP4 (Kabashima et al., 2003, Luft et al., 2002, Scandella et al., 2002). Because IL-13 stimulates the production of PGE_2_ by DCs and macrophages (Legler et al., 2006, Rey et al., 1999), it is possible that this is the mechanism by which IL-13 promotes DC migration to the draining LN. The effects of a EP4 agonist and antagonist on DC migration in vitro, as well as EP4 upregulation, and PGE_2_ production by IL-13 treatment further supported the notion that IL-13 promotes CCR7^+^ DC migration, in part through its influence on the EP4-PGE_2_ pathway.

It is well established that tissue DCs encountering antigens migrate to draining LNs, where they present antigens to naive T cells (Randolph et al., 2005). The current study also highlighted the importance of LNs as the site of Th2 cell differentiation. Repeated intranasal papain treatment induced much weaker allergic lung inflammation in *Rag2^−/−^Il2rg^−/−^* mice transplanted with WT BM cells than that in irradiated congenic WT recipient mice. We have previously shown that the former mice have normal ILC2s and other lymphocytes (Halim et al., 2012b). We believe that the observed differences are due to the lack of LNs in the former mice. Although papain treatment activated lung DCs, antigen presentation within the lung appeared to be very inefficient compared to antigen presentation in the draining mLN, and the migration of activated DCs into the mLN was critical for efficient activation of naive CD4^+^ T cells.

It remains to be determined whether CD40 on DCs is directly responsible for the differentiation of naive CD4^+^ T cells into Th2 cells in papain-treated mice. Studies with CD40-deficient DCs show that the interaction between CD40 and its ligand (CD154) is required for Th2, but not Th1, cell responses (MacDonald et al., 2002). However, CD154 expression on CD4^+^ cells is not essential for Th2 cell differentiation (Jenkins et al., 2008). Several other costimulatory receptor-ligand combinations, including ICOS-ICOSL (Tafuri et al., 2001) and OX40-OX40L (Jenkins et al., 2007), are also known to be involved in Th2 cell differentiation. However, how signals generated by these costimulatory receptor-ligand interactions in naive CD4^+^ T cells lead to GATA3 activation, which is critical for Th2 cell differentiation (Paul, 2010), are currently unknown. While the precise mechanisms by which Th2 cell differentiation is initiated in the mLN of papain-treated mice is still unclear, it should be noted that papain treatment or IL-13 administration does not induce IL-12p40 or IL-12p35 expression, which is thought to be critical for Th1 cell differentiation. IL-12p40 neutralization also had no effect on papain induced DC migration (data not shown).

We have also demonstrated that ILC2 activation and Th2 cell differentiation in papain-treated mice was IL-33-dependent. IL-33, which is considered to be an alarmin, is constitutively expressed in the nuclei of airway epithelial cells and rapidly released upon epithelial cell damage. Papain, but not heat-inactivated papain, activated ILC2s in WT mice but not IL-33-deficient mice, indicating that the protease activity of papain likely caused epithelial cell damage and IL-33 release, which in turn activated ILC2s. Indeed, intranasal administration of IL-33 directly stimulated ILC2s to produce IL-13, and coadministration of OVA and IL-33 promoted OVA-specific OT-II T cell differentiation into the Th2 cell pathway. These results suggested that any antigens that cause airway epithelial cell damage and/or activation and induce IL-33 release, and subsequent activation of ILC2s, could be potential allergens. Thus, the cascade of cell activation and cytokine production, initiated by airway epithelial IL-33 release, IL-13 production by ILC2s and downstream Th2 cell differentiation, type 2 cytokine production and IgE production is likely a common pathway of allergic lung inflammation in response to a broad range of allergens. Indeed, we also observed reduced Th2 cell differentiation in responses to both HDM extract and *Aspergillus sp.* protease allergens in the absence of ILC2s.

In summary, this study has revealed a critical role of ILC2s in the differentiation of naive CD4^+^ T cells into Th2 cells in response to protease allergens in the lung, thus providing an important clue to the long-standing question of why airway allergens induce an adaptive Th2 cell response. Our results redraw the map of type 2 immunity, placing ILC2s at the center of a common pathway of the Th2 cell cascade for a range of allergens. As one of the early and critical “domino tiles,” ILC2s likely exert a profound effect on Th2 cell-mediated inflammatory diseases such as asthma.

## Experimental Procedures

### Mice

C57Bl/6 (B6), B6-^Tg(TcraTcrb)425Cbn^/J (OT-II), B6.Pep3b, and B6.*Il33*^*−/−*^ (KOMP) mice were maintained in the BCCRC animal facility, and B6.*Il13*^egfp/egfp^ were maintained in the MRC ARES animal facility, under SPF conditions. B6.*Il4*^tm1Nnt^/J, B6.129S7-*Rag1*^tm1Mom^/J and B6.Cg-*Rora*^sg^/J mice were purchased from the Jackson Laboratories. B6*.Rag2*^*−/−*^*Il2rg*^*−/−*^ mice were purchased from Taconic Farms. Mice were used at 4–8 weeks of age. All animal use was approved by the animal care committee of the University of British Columbia in accordance with the guidelines of the Canadian Council on Animal Care or the UK Home Office.

### Bone Marrow Transplantation

B6*.Rag2*^*−/−*^*Il2rg*^*−/−*^ or B6.Pep3b mice were lethally irradiated (10 Gy), followed by intravenous transplantation of 10^7^ whole bone-marrow cells from 3- to 4-week-old mice. Mice were given ciprofloxacin and HCl in drinking water for 4 weeks, and used for analysis at 8–16 weeks posttransplant.

### Primary Leukocyte Preparation

Cell suspensions were prepared from the lungs, spleens, LNs, or BM as previously described (Veinotte et al., 2008).

### Intracellular Staining

Intracellular staining for GATA3 was performed with the Foxp3 intracellular staining kit (eBioscience) according to the manufacturer’s protocol. Intracellular staining for IL-4, IL-5, IL-13, and IFN-γ was performed with the Cytofix/Cytoperm kit (BD Biosciences) after 3 hr restimulation of 2 × 10^6^ total live nucleated cells in 500 μl RPMI-1640 media containing 10% FBS, Penicillin/Streptomycin (P+S), 2-mercaptoethanol (2-ME), Brefeldin A (GolgiPlug, BD Biosciences) or eBioscience protein transport inhibitor cocktail (for intracellular IL-4 detection), PMA (30 ng/ml), and ionomycin (500 ng/ml) at 37°C. Dead cells were excluded with eFluor® 450 or eFluor® 780 (eBioscience) fixable viability dye.

### Isolation of ILC2

Single cells were incubated with 2.4G2 to block Fc receptors and then stained with eFluor® 450-conjugated lineage marker mAbs (CD3ε, CD19, B220, NK1.1, Mac-1, GR-1, and Ter119), APC-eFluor® 780-conjugated B220, PE-conjugated CD127, PerCP-Cy5.5-conjugated CD25, PE.Cy7-conjugated Sca-1, and APC-conjugated CD117, V500-conjugated CD45, FITC-conjugated T1/ST2, PI viability dye, and purified by FACS.

### Lung Explant Cultures

Lung explants were made by injecting mouse lungs with 2% low melting point agarose (in RPMI-1640, kept at 37°C) via intratracheal injection. Lungs were allowed to cool, after which they were dissected. Explants of 300 μm thickness were made using a vibratome (Leica). Explants were cultured in 5.0 μm pore-size hanging-cell-culture inserts (Millipore) in RPMI-1640 media (10% FCS, P+S, 2 ME). Papain (5 μg/ml), anti-IL13 (0.5 μg/ml), rIL-13 (10 ng/ml), EP4-agonist (5 μM), or EP4-antagonist (5 μM) was added to the media. CCL21 or rmIL-13 (100 ng/ml) was added to the bottom compartment of the trans-well culture. Explants were cultured at 37°C for 14 hr. All contents of the trans-well inserts (including explants) were harvested and made to single-cell suspension, followed by Percoll purification of cells. All migrated cells in the bottom compartment were harvested. Cells were FcR-blocked and stained with PI, anti-CD11c, MHCII, B220, and CD45, followed by analysis by flow cytometry for DCs. Total number of cells was calculated with CountBright beads (Invitrogen). The percent of migrated DCs in each culture was calculated.

### In Vivo Stimulation

Mice were anesthetized by isofluorane inhalation, followed by the intranasal administration of rmIL-13 (1 μg), rmIL-33 (0.5 μg), OVA (50 μg), DQ-OVA (50 μg), house dust mite extract (100 μg), *Aspergillus oryzae* protease allergen (10 μg), papain, or heat-inactivated papain (10 μg) in 40 μl of PBS. Cultured ILC2s were adoptively transplanted (10^5^ cells) on days 0 and 1 by tail vein injection. Mice were sacrificed at indicated times, and spleens, pLNs, lungs, mLNs, and BAL (1 ml PBS) were collected or airways were instilled with 50:50 Tissue-Tek® O.C.T. Compound (Adwin Scientific) and PBS and fixed in formalin.

### OT-II Adoptive Transplant

OT-II cells were purified by CD4^+^ negative selection (StemCell Technologies) from OT-II mouse spleen. CD4^+^ OT-II cells were counted and labeled with CFSE (Invitrogen). On day 0, recipient mice were injected with 1 × 10^6^ CFSE labeled OT-II cells by tail vein injection.

### Statistics

Data were analyzed with GraphPad Prism 6 (GraphPad Software). A Student’s t test was used to determine statistical significance between two groups, and ANOVA was performed for multivariable analysis, with p ≤ 0.05 being considered significant.

## Author Contributions

T.Y.F.H. designed and performed the experiments and wrote the paper. I.M.G., C.A.S., and L.M. performed the experiments. M.J.G. designed and performed the experiments. A.N.J.M. and K.M.M. designed experiments and reviewed the paper. F.T. supervised the project, designed the experiments, and wrote the paper. C.A.S. and L.M. contributed equally to this work.

## Supplemental Information

Document S1. Figures S1–S6 and Supplemental Experimental Procedures
