# Supplementary material for: Group 2 Innate Lymphoid Cells Are Critical for the Initiation of Adaptive T Helper 2 Cell-Mediated Allergic Lung Inflammation
Source: Immunity. 2014 Mar 20;40(3):425–35. doi: 10.1016/j.immuni.2014.01.011 (PMC4210641; doi:10.1016/j.immuni.2014.01.011)
Supplement: Document S1. Figures S1–S6 and Supplemental Experimental Procedures [file mmc1.pdf]

Immunity, Volume *40*

Supplemental Information

Group 2 Innate Lymphoid Cells Are Critical for the Initiation of Adaptive T Helper 2 Cell-Mediated Allergic Lung Inflammation

Timotheus Y.F. Halim, Catherine A. Steer, Laura Mathä, Matthew J. Gold, Itziar Martinez-Gonzalez, Kelly M. McNagny, Andrew N.J. McKenzie, and Fumio Takei

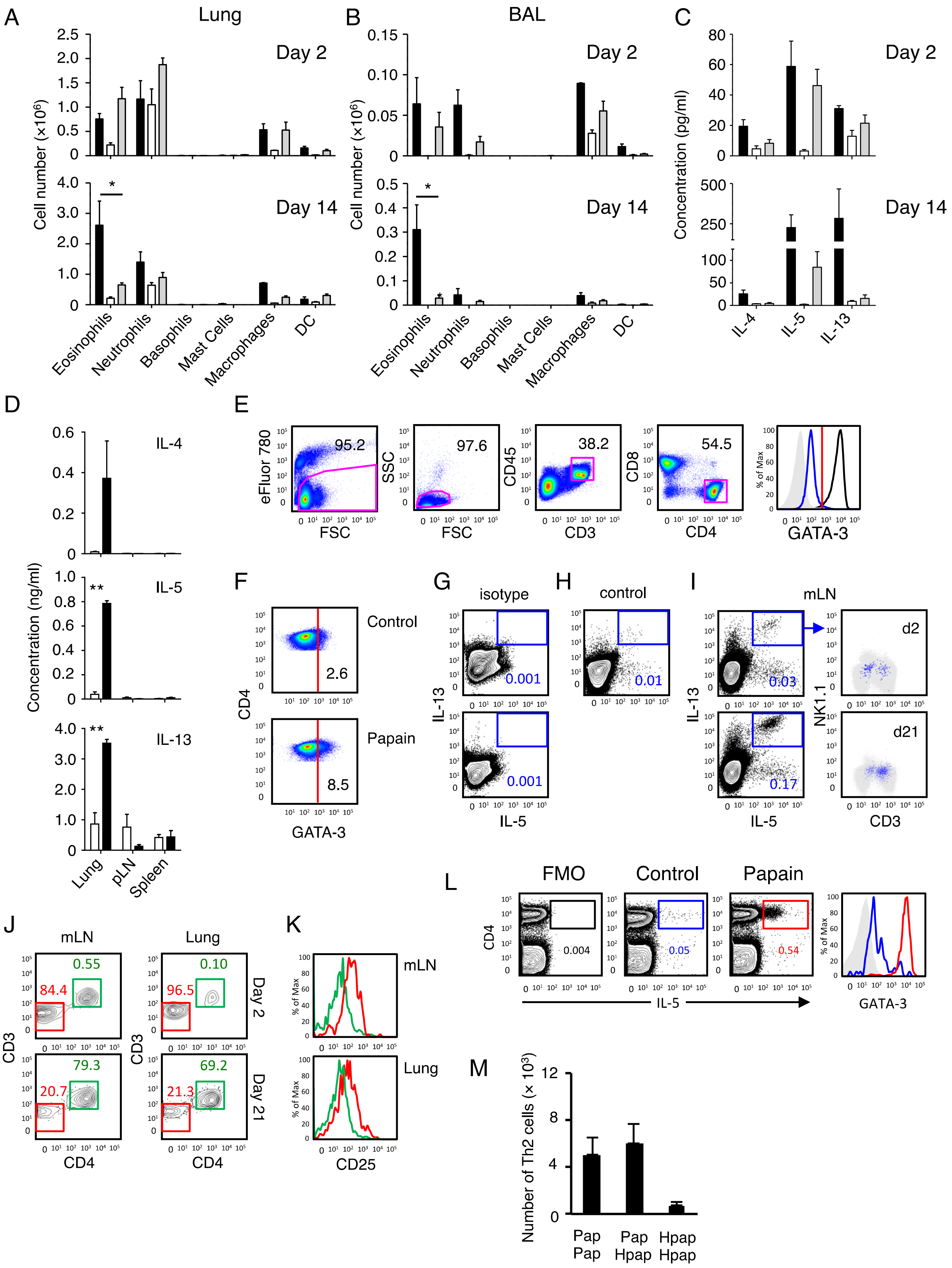
A
Lung
Day 2
Day 14
Cell number (×10⁶)
*
Eosinophils
Neutrophils
Basophils
Mast Cells
Macrophages
DC
B
BAL
C
Concentration (pg/ml)
IL-4
IL-5
IL-13
D
Concentration (ng/ml)
**
Lung
pLN
Spleen
E
eFluor 780
FSC
SSC
CD45
CD3
CD8
CD4
GATA-3
95.2
97.6
38.2
54.5
F
Control
Papain
2.6
8.5
G
isotype
H
control
I
mLN
IL-13
IL-5
0.001
0.01
0.03
0.17
NK1.1
d2
d21
J
84.4
0.55
96.5
0.10
20.7
79.3
21.3
69.2
Day 2
Day 21
K
CD25
L
FMO
0.004
0.05
0.54
M
Number of Th2 cells (× 10³)
Pap
Hpap

Figure S1, related to Figure 1

(**A-C**) Induction of protease mediated Th2 cell-type inflammation in *Rag1*$^{-/-}$ mice. WT (■) or T/B/NKT cell deficient *Rag1*$^{-/-}$ (■) mice injected with papain or control (□) as described in Figure 1A were sacrificed on days 2 and 14, followed by quantification of myeloid populations in the lung (**A**) and bronchoalveolar lavage (**B**) using flow cytometry. Cytokine levels in bronchoalveolar lavage were quantified by ELISA (**C**).

(**D**) Induction of Th2 cells after papain stimulation as measured by *in vitro* re-stimulation. Lymphocytes from indicated tissues were re-stimulated with immobilized anti-CD3ε and anti-CD28, followed by analysis of cytokine production by ELISA.

(**E-F**) Intracellular GATA3 analysis of $CD4^+$ T cells. Intracellular GATA3 staining was performed to assay the induction of Th2 cells. Briefly, to establish control staining, naïve $CD4^+$ T cells were harvested from WT mouse spleen, followed by *in vitro* culture in Th2-skewing conditions. Cells were harvested on day 5 and stained with fixable viability dye (eFluor780) and for cell-surface CD45, CD3, CD8 and CD4, followed by fixation/permeabilization and intracellular staining with isotype control or anti-GATA3 mAb. Live ($eFluor780^-$) SSC/FSC^{low} $CD45^+CD3^+CD4^+$ T cells were analyzed for intracellular GATA3. *In vitro* differentiated Th2 cells were used as a positive control (black), while isotype stained cells were used as non-specific staining control (blue). Grey histogram indicates fluorescence minus one (FMO) control (**E**). Day 21 papain stimulated and control mouse lungs and mLN (shown) were harvested, followed by immediate intracellular staining for GATA3 in $CD4^+$ T cells (**F**).

(**G-L**) Identification of IL-5 and IL-13 producing cells in the lung and mLN by intracellular staining. Lung or mLN cells from papain or control treated WT mice were harvested on day 2 or 21, followed by re-stimulation with PMA and ionomycin in the presence of Brefeldin A for 3

hours. Cells were subsequently stained with fixable viability dye and mAbs for cell surface markers (CD45, CD3, CD4, CD19, NK1.1 and CD25), followed by fixation/permeabilization and staining with isotype mAbs or anti-IL-5 and anti-IL-13 mAbs and analysis by flow-cytometry. Isotype stained live (eFluor780$^-$) CD45$^+$ cells were used to determine background staining in lung (top) and mLN (bottom) and set positive gates (**G**). Live CD45$^+$ Lung cells from day 2 and day 21 control mice were analyzed for percent IL-5$^+$IL-13$^+$ cells (**H**), which were electronically gated and analyzed for CD3 and NK1.1 expression. Live CD45$^+$ mLN cells from day 2 and day 21 mice were analyzed for percent IL-5$^+$IL-13$^+$ cells, which were subsequently electronically gated and analyzed for CD3 and NK1.1 expression (**I**). Subsequent analysis of IL-5$^+$IL-13$^+$ cells (which are NK1.1$^-$) shows that CD3$^+$ cells are also CD4$^+$ (green), whereas CD3$^-$ cells are CD4$^-$ (red) (**J**). NK1.1$^-$CD3$^-$CD4$^-$IL-5$^+$IL-13$^+$ cells (red) express higher levels of CD25 (**K**). Re-stimulated LN CD4$^+$ T-cells from papain or control injected mice on day 21 were analyzed for intracellular IL-5. IL-5$^+$CD4$^+$ T-cells were subsequently analyzed for GATA3 expression. Grey histogram indicates FMO control of CD4$^+$ T cells (**L**).

(**M**) Mice were sensitized on days 0 and 1, and challenged on day 13 with papain (pap) or heat-inactivated papain (Hpap) (top row). On day 20, mice were challenged with papain or Hpap (bottom row), followed by quantification of IL-5$^+$IL-13$^+$ CD3$^+$CD4$^+$ Th2 cells in the lung.

Data are representative of at least 3 independent experiments. Mean ± S.E.M. in A-D and M. Number represents percent gated cells in dot plots and histograms. * = $p < 0.05$ ** = $p < 0.01$ *** = $p < 0.001$ (two-tailed Student's t-test).

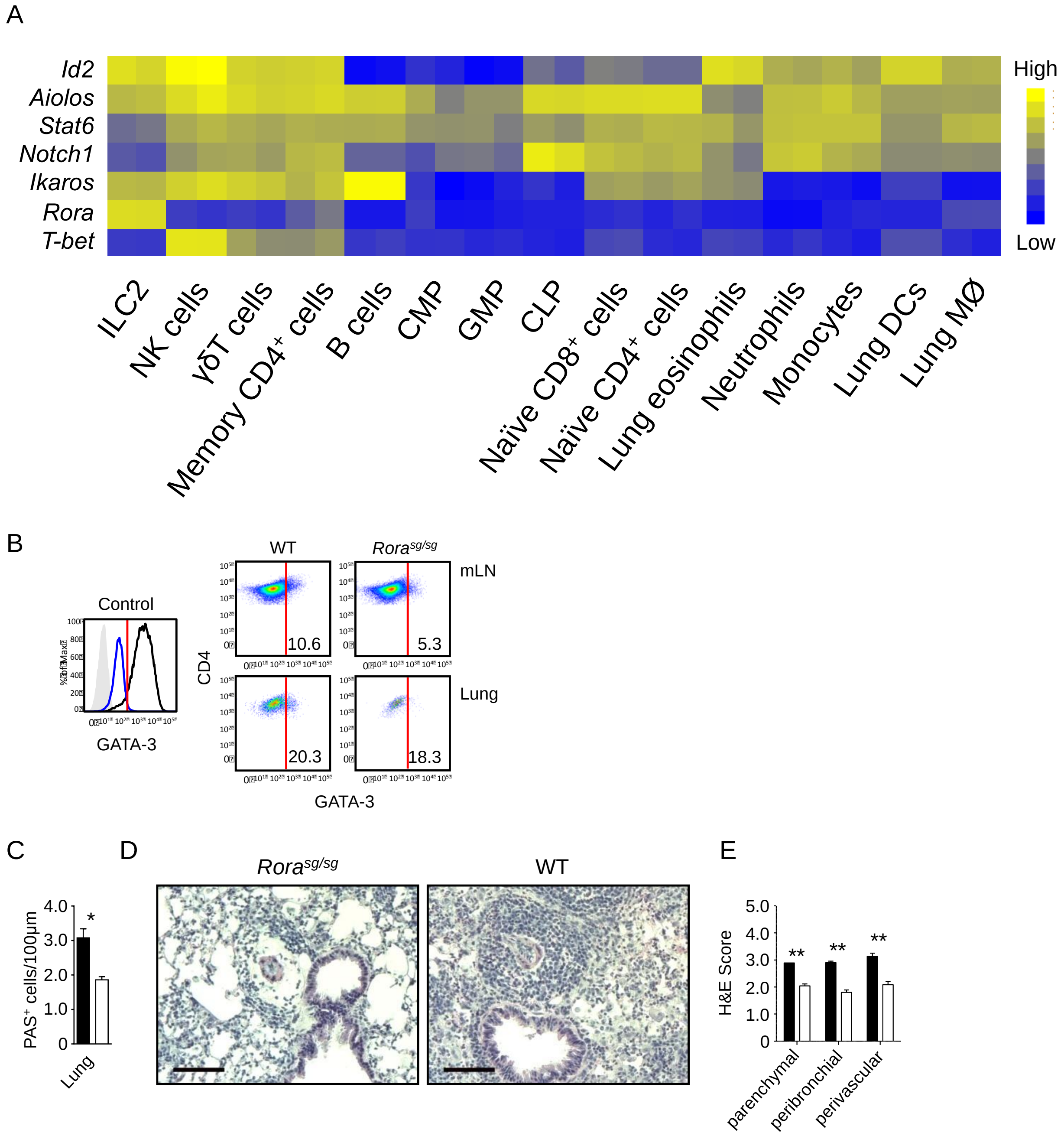

Figure S2, related to Figure 2

(**A**) Microarray analysis of different myeloid and lymphoid progenitors, and mature subsets for gene expression. Naïve WT lung ILC2 gene expression (Halim et al, 2012a) was compared to publicly available (ImmGen) gene expression microarray datasets of common myeloid

progenitors (CMP), granulocyte-macrophage precursor (GMP), common lymphoid progenitors (CLP), and other mature myeloid and lymphoid lineages.

(**B**) GATA3 intracellular staining of *ex vivo* $CD4^+$ T cells in the mLN and Lung of WT and $\textit{Rora}^{sg/sg}$ BMT mice analyzed on day 21. Experiment was performed as described in Figure S1E.

(**C-E**) ILC2 cell deficient $\textit{Rora}^{sg/sg}$ BMT mice show significantly less mucus hyperproduction and inflammation. Lungs of papain stimulated WT or $\textit{Rora}^{sg/sg}$ BMT mice were infused with O.C.T. compound and fixed in formalin, followed by sectioning and periodic acid-Schiff (PAS) or H&E (**D**) staining. Samples were blinded and sent to an expert for histological scoring of mucus overproduction (**C**) or inflammation (**E**).

Scale bar indicates 100 μm, data are representative of at least 3 independent experiments. Numbers in dot-plot represent percent of gated cells. Mean ± S.E.M. shown in C and E, * = $p < 0.05$ ** = $p < 0.001$ (two-tailed Student’s t-test).

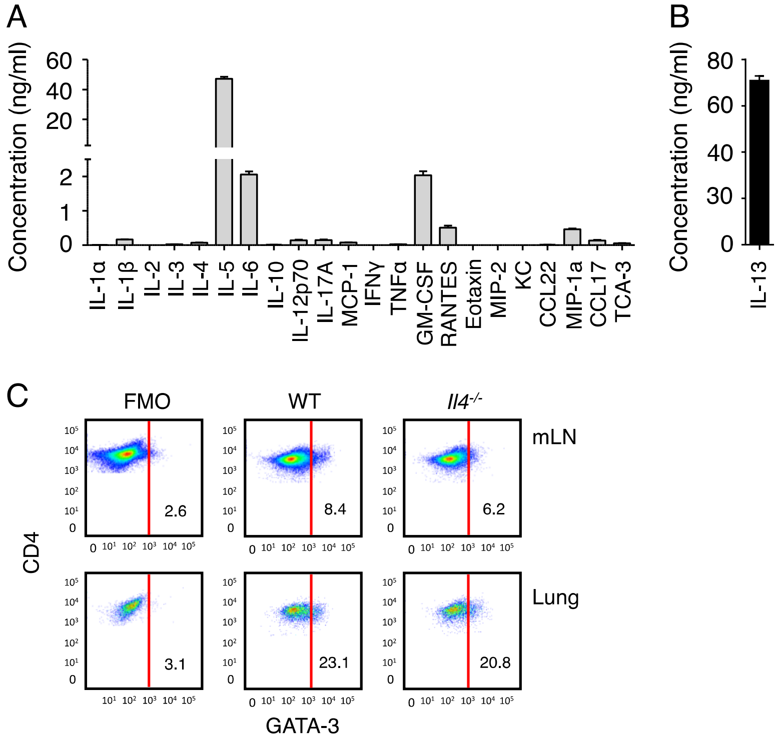

Figure S3, related to Figure 3

(**A-B**) Cytokine production by *in vitro* stimulated ILC2s. FACS purified lung ILC2 (1000 cells) were stimulated *in vitro* with IL-33 (10 ng/ml) plus TSLP (10 ng/ml) for 7 days, after which culture supernatant was harvested and analyzed for cytokine and chemokine concentration by multiplex (**A**) or ELISA (**B**).

(**C**) Intracellular GATA3 staining in *Il4*$^{-/-}$ and WT mice was performed as described previously.

Data are representative of 4 independent experiments. Numbers in dot-plot represent percent of gated cells. Mean ± S.E.M. shown in A and B.

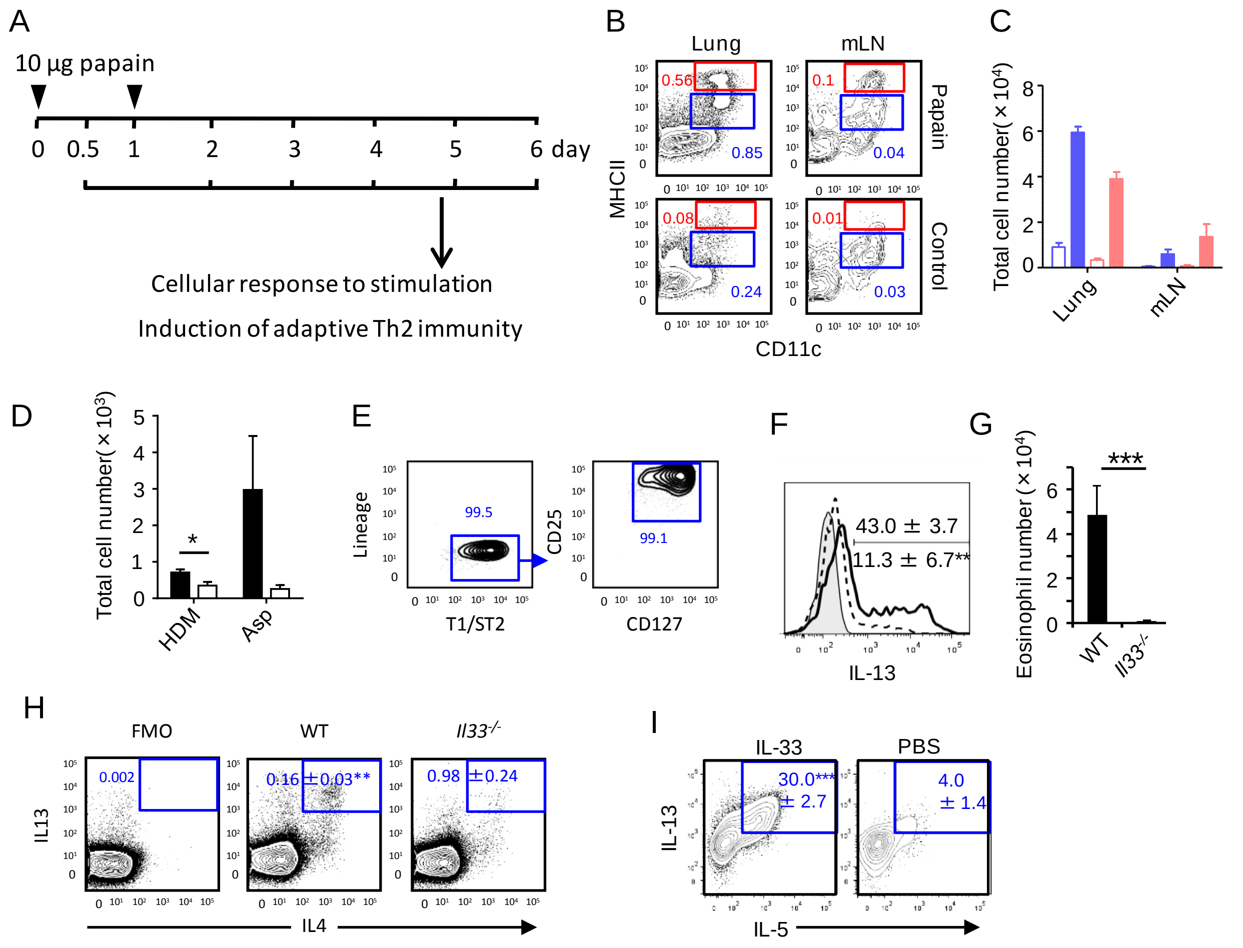

Figure S4, related to Figure 4

(A) We performed a time-course experiment to analyze the kinetics of the immune response leading up to induction of adaptive Th2 cell immunity in the lungs of WT mice.

(B-C) Papain induces the maturation and migration of DC. Mice treated with papain or control were analyzed on day 2 for DC activation in the lung or mLN (**B**). Percent of activated CD11c^{+}MHCII^{hi} (red) and non-activated CD11c^{+}MHCII^{lo} (blue) amongst total live cells is shown. Absolute numbers of activated and non-activated DCs in papain treated (solid) or control (clear) lung and mLN were measured (**C**).

(**D**) Analysis of alternative allergens. WT (■) and *Rora*$^{sg/sg}$ (□) BMT mice were injected on days 0 and 1 with house dust mite (HDM) extract or *Aspergillus oryzae* protease allergen (Asp). The mLN cells were harvested on day 6 and stained for intracellular IL-5 and IL-13. Total numbers of IL-5^{+}IL-13^{+} CD4^{+} T cells were quantified.

(**E**) *In vitro* cultured ILC2s were analyzed for purity by flow cytometry before add-back experiments. Live (PI-) CD45^{+} cells were electronically gated and analyzed for expression of lineage markers CD127, T1/ST2 and CD25.

(**F-G**) Impaired ILC2-stimulation and inflammation in papain-treated IL-33-deficient mice. WT and IL-33-deficient mice received intranasal papain administration on days 0 and 1, and lung ILC2s were analyzed by flow-cytometry on day 2. Histograms show **i**ntracellular IL-13 staining of WT (solid line) and IL-33-deficient (broken line) ILC2s with FMO (shaded) (**F**). Eosinophils in the BAL prepared from the treated mice were analyzed by flow-cytometry, and the total cell numbers were calculated and shown in bar graph in (**G)**.

(**H**) Impaired Th2 cell generation in papain-treated IL-33-deficient mice. CD4^{+}CD3^{+} T cells in the mLN of papain-treated WT and IL-33-deficient mice were analyzed on day 6 for intracellular IL-4 and IL-13 by flow-cytometry. The numbers indicate the percentages (mean ± SEM) of IL-4^{+}IL-13^{+} cells among CD4^{+} T cells.

(**I**) Stimulation of ILC2s by intranasal IL-33. WT mice were treated on days 0 and 1 with intranasal injections of rmIL-33 or PBS, and lung ILC2s were analyzed for intracellular IL-5 and IL-13 on day 3. The numbers indicate the percentages (mean ± SEM) of IL-5^{+}IL-13^{+} cells among ILC2s.

Data are representative of 3 independent experiments. Numbers in dot-plot represent mean percent of gated cells. Mean ± S.E.M. shown in C, D, G and H, * = $p < 0.05$ *** = $p < 0.001$ (two-tailed Student's t-test).

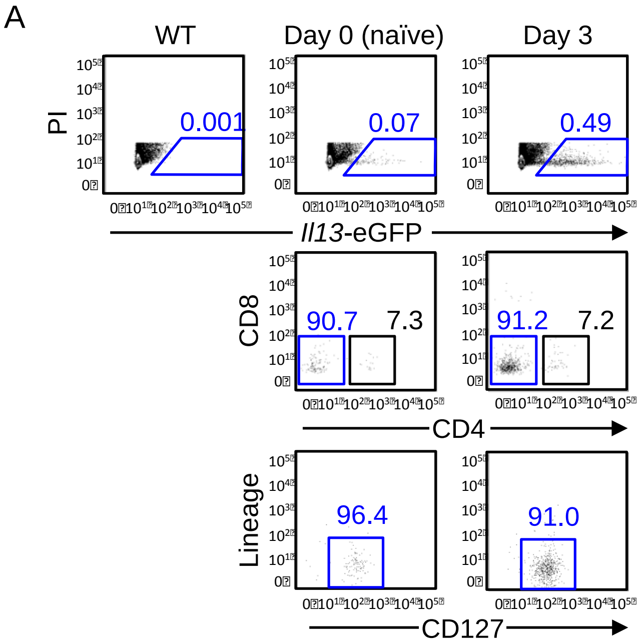

Figure S5, related to Figure 5

(**A**) Analysis for *Il13*^{egfp}-positive cells in naïve and papain-treated mice. Lungs of untreated (naïve) *Il13*$^{egfp/+}$-mice, or papain treated WT (control) and *Il13*$^{egfp/+}$ mice were analyzed for eGFP-expression. eGFP^{+} cells were identified as CD3^{+}CD4^{+} T cells or CD4^{-}CD8^{-}CD3^{-}Lineage^{-} CD127^{+} ILC2 by flow-cytometry. Papain treated mice were injected on day 0 and 1, followed by analysis on day 3.

Data are representative of 3 independent experiments.

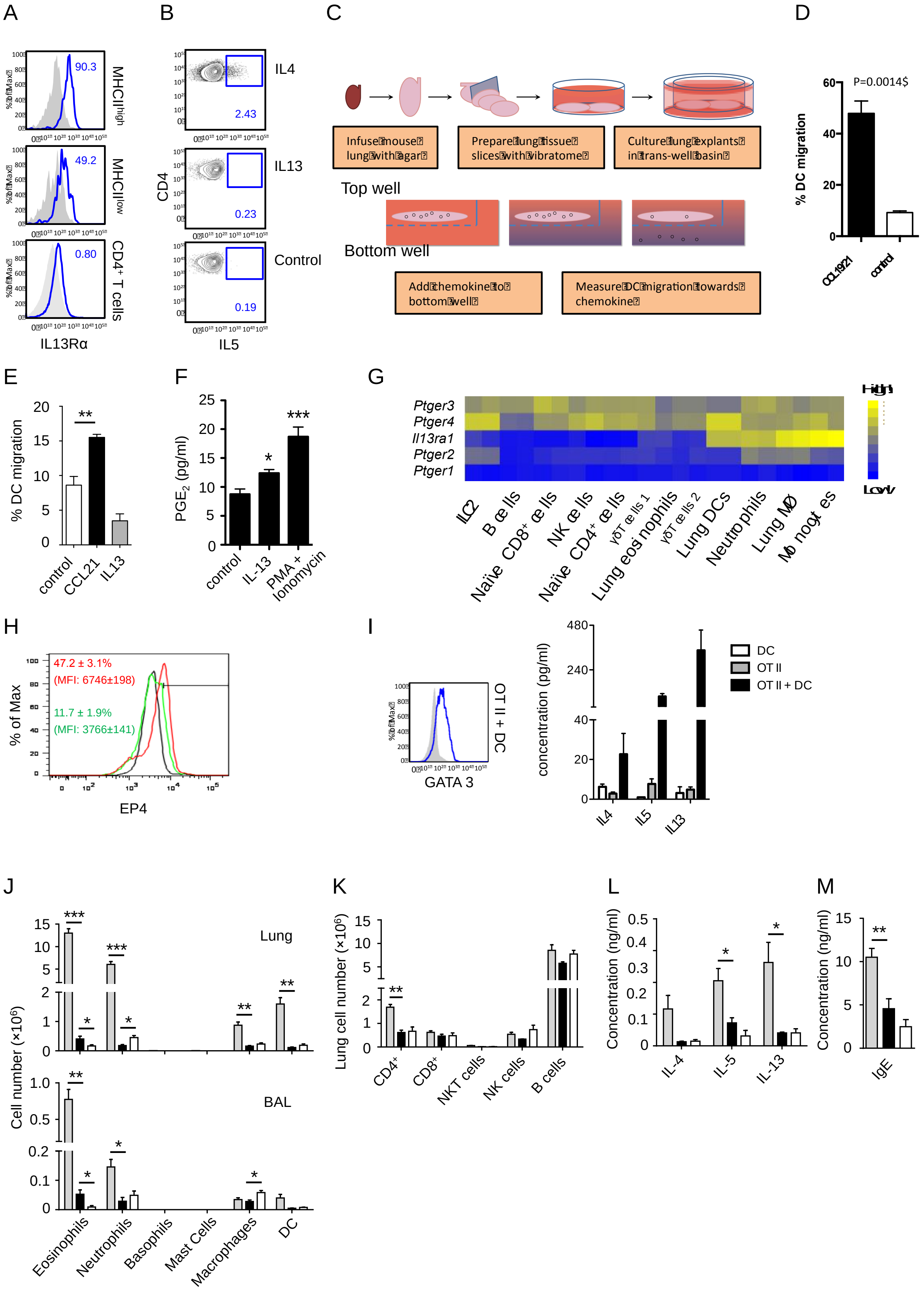

A
90.3
MHCII high
49.2
MHCII low
0.80
CD4+ T cells
IL13Rα
B
IL4
2.43
IL13
0.23
Control
0.19
CD4
IL5
C
Infuse mouse lung with agar
Prepare lung tissue slices with vibratome
Culture lung explants in trans-well basin
Top well
Bottom well
Add chemokine to bottom well
Measure DC migration towards chemokine
D
P=0.0014$
% DC migration
CCL19/21
control
E
% DC migration
**
control
CCL21
IL13
F
PGE2 (pg/ml)
*
***
control
IL-13
PMA + Ionomycin
G
Ptger3
Ptger4
Il13ra1
Ptger2
Ptger1
High
Low
H
% of Max
47.2 ± 3.1%
(MFI: 6746±198)
11.7 ± 1.9%
(MFI: 3766±141)
EP4
I
OT II + DC
GATA 3
concentration (pg/ml)
DC
OT II
OT II + DC
IL4
IL5
IL13
J
Lung
BAL
Cell number (×10⁶)
Eosinophils
Neutrophils
Basophils
Mast Cells
Macrophages
DC
K
Lung cell number (×10⁶)
CD4+
CD8+
NKT cells
NK cells
B cells
L
Concentration (ng/ml)
IL-4
IL-5
IL-13
M
Concentration (ng/ml)
IgE

Figure S6, related to Figure 6

(**A-B**) IL-13Rα analysis. WT mouse DCs and $CD4^+$ T cells were analyzed for IL-13Rα cell surface expression by flow cytometry (**A**). Purified naïve $CD4^+$ T cells were cultured *in vitro* with IL-4 or IL-13 to induce Th2 cell differentiation. Cultures were harvested on day 6 and cells were analyzed by intracellular staining for IL-5 (**B**).

(**C-D**) Lung explants were prepared from agar-injected lungs, and cultured in trans-well inserts in 6-well tissue culture dishes. Bottom compartments were spiked with chemokines or PBS control, creating a gradient. DC trans-well migration was measured after 14 hours by collecting the entire top and bottom well content, followed by flow cytometric analysis for $CD11c^+MHCII^+$ DCs. Absolute DC numbers were determined using CountBright beads, followed by subsequent calculation of percent DC migration (**D**).

(**E**) Naïve WT mouse lung explants were stimulated with papain *ex vivo*, cultured in trans-well inserts, and exposed to a CCL21, IL-13, or PBS (control) gradient as in (**C**). The percent of DC migration was calculated after 14 hours.

(**F**) Whole lung leukocytes (1.5×10^5 cells in 200 μl media) were stimulated with PBS (control), IL-13 (10 ng/ml), or PMA + ionomycin. Culture supernatants were analyzed at 24h for PGE_2 concentration.

(**G**) Microarray analysis for gene expression. Naïve WT lung ILC2 gene expression (Halim et al, 2012a) was compared to publicly available (ImmGen) gene expression microarray datasets of mature myeloid and lymphoid lineages.

(**H**) EP4 detection by flow-cytometry. Mice were treated with PBS (green) or IL-13 (red) via intranasal injection, followed by analysis of lung cells the next day. Lung leukocytes were stained for CD11c, CD11b and eFluor® 450 viability dye, fixed, permeabilized and stained with

anti-EP4 antibody and PE-conjugated secondary antibody (anti-mouse IgG). Live lung DCs ($CD11b^+CD11c^+$) were gated and analysed for EP4 expression. FMO (no primary antibody) was used to determine negative staining (black). MFI and percent positive cells were calculated (n=6 per group).

(**I**) Papain stimulated DCs are sufficient for Th2 cell differentiation of OT-II cells. WT mice were treated with two daily intranasal injection of papain + DQ-OVA (50µg), and $CD11c^+CD11b^+DQ\text{-}OVA^{hi}$ DCs were purified from the mLN by cell sorting on day 4. $CD4^+$ T cells were isolated from naive OT-II mice and $2x10^5$ were incubated with or without DCs ($1x10^3$) in 200 µl_media with 0.3µM OVA peptide for 5 days. GATA-3 expression in OT-II T cells was analyzed by intracellular staining and flow-cytometry. Shaded histogram shows FMO. Cytokines in the culture supernatants were analyzed by ELISA.

(**J-M**) LN-deficient *Rag2$^{-/-}$Il2rg$^{-/-}$* BMT mice analysis. WT BMT into Pep3b (■), or WT (■) and *Rora$^{sg/sg}$* (□) BMT into *Rag2$^{-/-}$Il2rg$^{-/-}$* mice were treated with papain and analyzed on day 21 for myeloid cell numbers in the lung and BAL (**J**). Lymphoid cell numbers were measured in the lung (**K**). The BAL was analyzed for concentrations of cytokines (**L**). Serum IgE concentration was measured (**M**).

Figure Data are representative of at least 3 independent experiments. Numbers in dot-plot represent mean percent of gated cells. Mean ± S.E.M. shown in D-F, J-m, * = $p < 0.05$ ** = $p < 0.01$ *** = $p < 0.001$ (two-tailed Student's t-test).

Supplemental Experimental Procedures

Antibodies, reagents and flow-cytometers. FITC-conjugated anti-CD3ε, CD19, B220, NK1.1, Mac-1, Gr-1, Ter119, CD45.2, Phycoerythrobilin (PE)-conjugated streptavidin, anti-CD3ε, CD127, CD40, PerCP-Cy5.5-conjugated anti-CD19, CD25, NK1.1, CD3ε, CCR7, PE.Cy7-conjugated anti-Sca-1, DX5, CD4, Allophycocyanin (APC)-conjugated anti-CD117, IL-4, CD8, CD25, FcεR1α, APC-eFluor780®-conjugated anti-B220, Alexa Fluor® 700-conjugated anti-CD45.1, CD11c, eFluor® 450-conjugated anti-CD3ε, CD19, B220, NK1.1, Gr-1, CD11b, Ter119, NKp46, and eFluor® 650NC-conjugated anti-CD4 and Thy1.2 were purchased from eBioscience. FITC-conjugated anti-7/4 was purchased from Abcam. FITC-conjugated anti-MHCII, IFNγ, PE-conjugated anti-IL-13, Siglec-F, APC-conjugated anti-IL-5 and BD Horizon V500-conjugated anti-CD45 and CD45.2 were purchased from BD Bioscience. FITC-conjugated anti-ST2 was purchased from MD Bioproducts. Anti EP4 antibody was purchased from Santa Cruz Biotechnology. Propidium iodide (PI), eFluor® 450 (eBioscience), eFluor® 780 (eBioscience) or DAPI reagents were used to exclude non-viable cells. Unconjugated anti-IL-13, rmIL-13, rmIL-33 and TSLP were purchased from eBioscience, E64, papain, PMA and ionomycin were purchased from Sigma Aldrich. DQ™-Ovalbumin (DQ-OVA) was purchased from Molecular Probes, Invitrogen. House dust mite extract was purchased from Greer Laboratories. *Aspergillus oryzae* protease allergen was purchased from Sigma. EP4-antagonist (L-161,982) and EP4-agonist (CAY10580) were purchased from Cayman Chemicals. Papain was heat-inactivated at 100°C for 1 hr. BD Fortessa, BD Calibur (Cytek 6 color upgrade) and Canto II were used for phenotypic analysis; BD FACS Aria II was used for cell sorting and phenotypic analysis. Flowjo v. 8.6 was used for data analysis.

Re-stimulation cultures and whole lung leukocyte cultures. Lymphocytes were obtained from tissues as described and counted for live nucleated cells, followed by culture of 2×10^6 cells in 500 µl RPMI-1640 media containing 10% FBS, penicillin and streptomycin (P+S), 2-Mercaptoethanol (2 ME) at 37°C for 72 hours. Re-stimulation cultures were stimulated with recall-antigen: papain (20 µg/ml) + E64 papain inhibitor (10 µM), heat-inactivated papain (20 µg/ml), or in pre-coated wells (αCD3ε (10 µg/ml) + αCD28 (1 µg/ml) mAb, eBioscience). Supernatant was analyzed for cytokine concentration at 72 hours. 1.5×10^5 cells/well (lung leukocytes) were cultured in 200 µl RPMI 10% FBS ± rmIL13 (10ng/ml), or PMA (30 ng/ml) + ionomycin (500 ng/ml) for 24 hours.

ILC2 *in vitro* culture. Immature ILC2 cells (Halim et al., 2012b) were FACS purified from WT bone-marrow and expanded in 200 µl RPMI-1640 media containing 10% FBS, P+S, 2 ME, IL-33 (10 ng/ml) and TSLP (10 ng/ml) at 37°C for 7 days. ILC2s were harvested, checked by flow-cytometry for phenotype, counted and injected (10^5 cells per mouse) into recipient mice.

Quantification of cytokine and Ig production. IgE (BD Bioscience), IFNγ, IL-4, IL-5, and IL-13 ELISAs (eBioscience) were performed according to the manufacturer's protocol. Cell-culture samples were analyzed by Q-Plex ELISA technology (Quansys Biosciences). PGE_2 concentrations were measured by competitive bioassay (Cayman Chemicals).

Gene expression analysis. We obtained microarray data sets for the listed cell-types from data assembled by the ImmGen consortium (Heng et al., 2008), which was compared to our own ILC2 microarray data (Halim et al., 2012a). Data analysis was performed with FlexArray 1.5 (Genome Quebec).

Lung inflammation analysis. Lung tissue was processed as described previously; lung and BAL cells were then counted and identified by flow-cytometry. Fixed tissues were embedded in paraffin and processed for H&E or PAS staining by the Centre for Translational and Applied Genomics (Vancouver, Canada). For histology, lung sections of three different depths per animal were blinded and sent to an unbiased expert for scoring (30 fields of view per animal).

Supplemental References

Heng, T.S., and Painter, M.W.; Immunological Genome Project Consortium. (2008). The Immunological Genome Project: networks of gene expression in immune cells. Nat. Immunol. *9*, 1091–1094.
